# Report of the Medical Department of the University of Pennsylvania, for the Session of 1851-2, to the Alumni of the School

**Published:** 1852-12

**Authors:** 


*Report of the Medical Department of the University of Pennsylvania, for the Session of* 1851–2, *to the Alumni of the School.* By THE MEDICAL FACULTY.

The medical reader who is conversant with the bulletins emanating from the Med. Department of the University of Penn., for several years past, mus t be surprised by the announcement of a reduction of the lecture term in that school to five months; but if the reader be at all given to critical analysis, a perusal of the document entitled as above, will excite astonishment aside from the fact just stated. As an argumentative effort it is one of the most curious productions that have ever fallen under our notice.

The faculty commence by professing to have been earnestly engaged in endeavors to "improve the system of medical education in the United States;" and that "one of the means by which it (their institution) has sought to accomplish this object has been the prolongation of the scholastic term of instruction." The report goes on to say, that this extension of the lecture term was made with the full appreciation of the risk of damaging the private interests of the faculty. It also declares, in the strongest terms, that the prolonged session has worked admirably so far as the interests of medical education are concerned. To quote its language, on the part of their students, "there has been a progressive improvement in all respects, increasing steadiness of deportment, attention to study and earnestness of self-improvement have been observable; and the examinations, though not less strict than formerly, have each year resulted in relatively fewer failures to attain to the honors of the institution." Such having been the results — the very results for which the extension of the term was adopted — why, the reader at once is ready to exclaim, in the name of consistency, is the plan not adhered to? The report furnishes the reply to this inquiry. Reading farther onward, it is in effect admitted that the reduction of the term is owing to the number of students having fallen off. The class has been reduced gradually from 509 to 410. The last named number is not large enough to meet the views of the faculty of the University of Pennsylvania. With this, in their view inadequate amount of patronage, and with the prospect of a *farther* depreciation, they hasten to return to the length of term which past experience has shown to be most profitable.

Now to this course, we concede at once, the critic has no right to take exceptions, save that he cannot well forbear to notice the incongruity of the professions which preceded the announcement. But in ignoring motives of self-interest, the report goes much farther than merely to claim that in extending the term of instruction the faculty were primarily actuated by a desire to improve medical education. It enunciates "two distinct principles upon which a medical school may be conducted; one having reference to the interests of the institution, the medical profession and the country; *the other to the temporary and pecuniary interests of the professors.*" We have italicized the portion of the foregoing quotation to which we would particularly call the reader's attention. The writer of the report proceeds to repudiate motives derived from the "temporary and pecuniary interests of the professors;" and, yet, the lengthened term is abandoned, by his own showing, wholly because, under this system, the number of students has decreased. The plain English of the matter is, that the school does not pay so well as with the shorter term. The faculty were willing to adopt the extended term so long as the aggregate amount of fees remained the same, but they are not disposed to stand any reduction in emolument; and rather than do this they proceed to reduce the length of the term, notwithstanding the long session has worked so admirably in behalf of the interests of medical education. No one has a right to complain of this change of policy by the Med. Dep. of the University of Pennsylvania. They are certainly under no obligation to diminish the incomes of the faculty for the sake of medical education; but the readers to whom this document is addressed, are justified in taking exceptions to the demand on their good sense when credit for the utmost disinterestedness is claimed on the one hand, and, on the other hand, motives of self-interest are virtually acknowledged.

But the curiosity of this document does not end here. The writer proceeds to state that owing to devoting the first week to introductories, the loss of a week at the holidays, and the examinations during the last two weeks of the term, there has, in reality, been only five months of solid teaching, while the six months' session was nominally in operation. And the report concludes by submitting abundant reasons why, with the proposed reduction to five months, as much instruction may be given, and with as much efficiency as heretofore! If we had space we would copy the portion of the report in which this branch of the subject is discussed. But we refer the reader to the report itself for the correctness of the assertion just made. Let him then compare the facts and views therein presented with the statement of the advantages of the six months' course which we have quoted near the commencement of this article! Let him contrast the arguments now advanced by the medical department of the University of Pennsylvania in favor of a *five* months' term with the position heretofore taken by that institution with respect to the extension of the term to six months and the claims which she has for years urged upon the profession for patronage on the score of the great advantages of the lengthened session! In this point of view the report affords scope enough for criticism. We have no disposition, however, to enter on the task of a more minute dissection, especially since this has been done most effectually by the scalpel of the caustic editor of the Western Journal of Medicine and Surgery — Dr. Bell.

